# Supplementary figures and images for: Free fatty acids and peripheral blood mononuclear cells (PBMC) are correlated with chronic inflammation in obesity
Source: Lipids Health Dis. 2023 Jul 4;22:93. doi: 10.1186/s12944-023-01842-y (PMC10318674; doi:10.1186/s12944-023-01842-y)

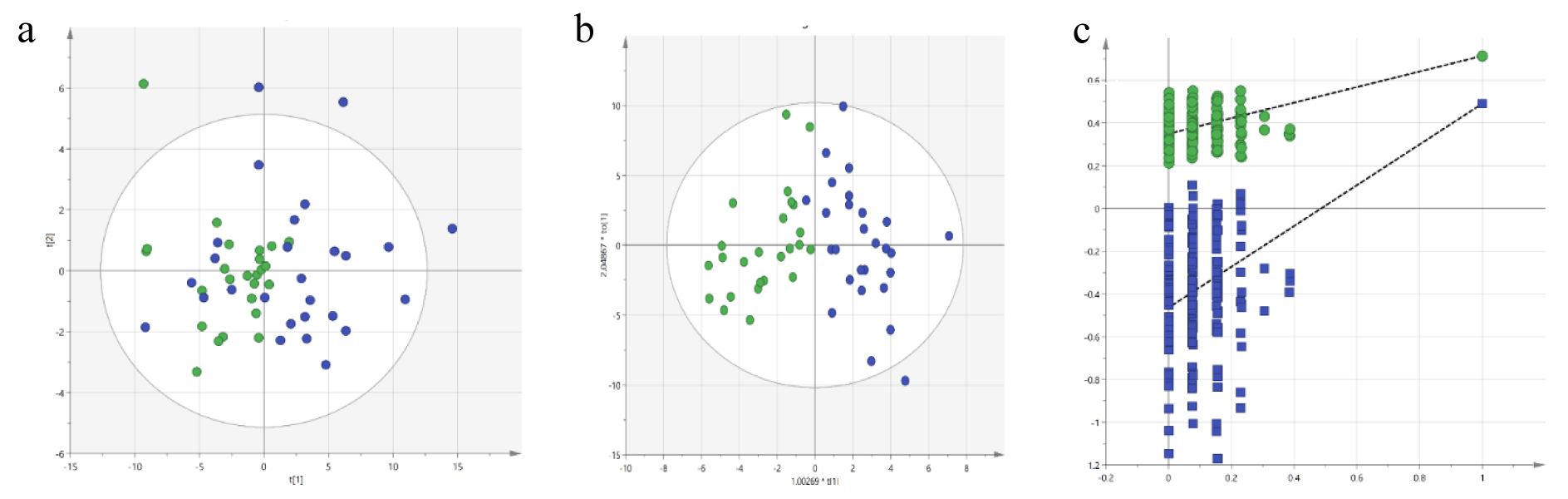

Supplement: Supplementary file 1 — Supplementary Fig. 1. Screening of potential biomarkers of obesity, (a) PCA (green: Normal weigh, blue: Obesity), (b) OPLS-DA (green: Normal weigh, blue: Obesity), (c) model of random array experiment (n: 200, green: R2, blue: Q2). [file 12944_2023_1842_MOESM1_ESM.tif]

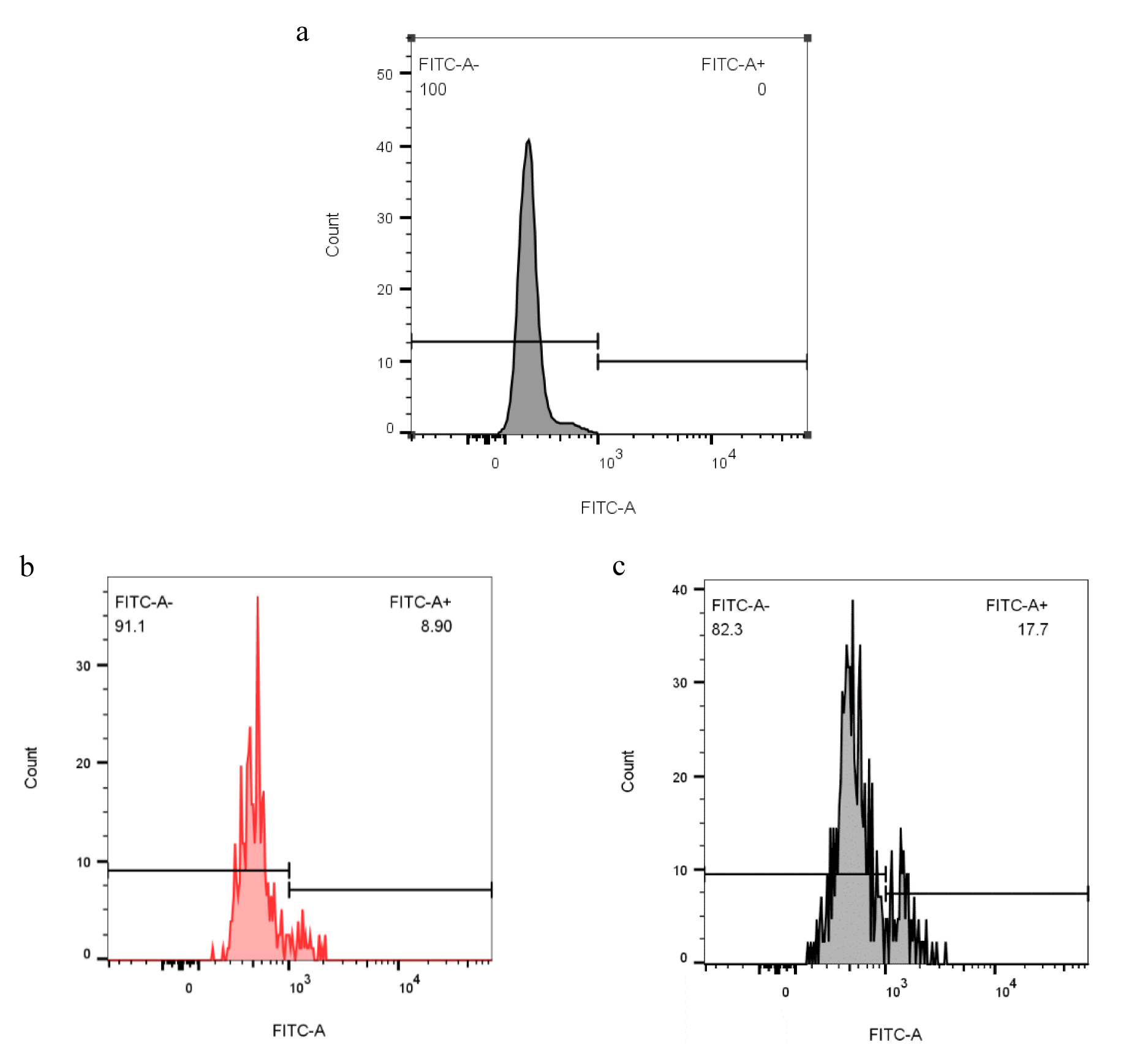

Supplement: Supplementary file 2 — Supplementary Fig. 2. Gating strategy. (a) use the control cells without antibody as the control, define the range of negative cells (NF- κB marked with FITC), (b) Proportion of NF-κB p65 positive monocytes in normal weight, and (c) Proportion of NF-κB p65 positive monocytes in obesity. PBMC subsets (Monocytes, Lymphocytes, Granulocytes) were determined by combining cell size and the number of cellular particles to determine their position, the gating strategy of NF-κB p65 is the same as that of CD36 and TLR4. [file 12944_2023_1842_MOESM2_ESM.tif]
