# Supplementary material for: Free fatty acids and peripheral blood mononuclear cells (PBMC) are correlated with chronic inflammation in obesity
Source: Lipids Health Dis. 2023 Jul 4;22:93. doi: 10.1186/s12944-023-01842-y (PMC10318674; doi:10.1186/s12944-023-01842-y)
Supplement: Supplementary file 3 — Supplementary Table 1. The fatty acids corresponding to the abbreviations. [file 12944_2023_1842_MOESM3_ESM.docx]

Supplementary Table 1. The fatty acids corresponding to the abbreviations

| Abbreviation for Fatty Acids | The full name of fatty acids | Types |
| --- | --- | --- |
| C4:0 | butyrate | SFA |
| C6:0 | hexanoate | SFA |
| C8:0 | octanoate | SFA |
| C10:0 | decanoate | SFA |
| C11:0 | undecanoate | SFA |
| C12:0 | dodecanoate | SFA |
| C13:0 | tridecanoate | SFA |
| C14:0 | myristate | SFA |
| C14:1N5 | myristoleate | MUFA |
| C15:0 | pentadecanoate | SFA |
| C15:1N5 | cis-10-pentadecenoate | MUFA |
| C16:0 | palmitate | SFA |
| C16:1N7 | palmitoleate | MUFA |
| C17:0 | heptadecanoate | SFA |
| C17:1N7 | cis-10-heptadecenoate | MUFA |
| C18:0 | stearate | SFA |
| C18:1TN9 | elaidate | MUFA |
| C18:1N9 | oleate | MUFA |
| C18:2TTN6 | linolelaidate | PUFA |
| C18:2N6 | linoleate | PUFA |
| C18:3N6 | γ-linolenate | PUFA |
| C18:3N3 | linolenate | PUFA |
| C20:0 | arachidate | SFA |
| C20:1N9 | cis-11-Eicosenoic acid ester | MUFA |
| C20:2N6 | cis-11,14-Eicosadienoic acid ester | PUFA |
| C21:0 | heneicosanoate | SFA |
| C20:3N6 | cis-8,11,14-Eicosatrienoic acid ester | PUFA |
| C20:4N6 | arachidonate | PUFA |
| C20:3N3 | cis-11,14,17-Eicosatrienoic acid ester | PUFA |
| C22:0 | behenate | SFA |
| C20:5 | cis-5,8,11,14,17-Eicosapentaenoic acid ester | SFA |
| C22:1N9 | erucate | MUFA |
| C22:2N6 | cis-13,16-Docosadienoic acid ester | PUFA |
| C23:0 | tricosanoate | SFA |
| C22:4N6 | docosatetraenoate | PUFA |
| C22:5N6 | docosapentaenoate | PUFA |
| C24:0 | tetracosanoate | SFA |
| C22:5N3 | docosapentaenoate | PUFA |
| C24:1N9 | cis-15-tetracosenoate | MUFA |
| C22:6N3 | cis-4,7,10,13,16,19-Docosahexaenoic acid ester | PUFA |
