# Supplementary material for: Free fatty acids and peripheral blood mononuclear cells (PBMC) are correlated with chronic inflammation in obesity
Source: Lipids Health Dis. 2023 Jul 4;22:93. doi: 10.1186/s12944-023-01842-y (PMC10318674; doi:10.1186/s12944-023-01842-y)
Supplement: Supplementary file 4 — Supplementary Material 4 [file 12944_2023_1842_MOESM4_ESM.pdf]

Peripheral blood mononuclear cells (PBMC) involved in obesity related chronic inflammation caused by free fatty acids

By Liqiang Su

WORD COUNT 4771

TIME SUBMITTED 08-MAY-2023 07:43PM

PAPER ID 99504501

Peripheral blood mononuclear cells (PBMC) involved in obesity related chronic inflammation caused by free fatty acids

Abstract: **Objective:** The biomarkers associated with obesity were identified from 40 fatty acids (FAs) in the blood, and analyze the relationship between the biomarkers and chronic inflammation. Furthermore, by analyzing the difference in the expression of CD36, TLR4 and NF-κB in peripheral blood mononuclear cells (PBMC) between obese and standard weight people, understand that immunophenotype PBMC is associated with chronic inflammation. **Methods:** This study is a cross-sectional study. Participants were recruited from the Yangzhou Lipan weight loss training camp from May 2020 to July 2020. The sample size was 52 individuals, including 25 in the normal weight group and 27 in the obesity group. Individuals with obesity and controls of normal weight were recruited to identify biomarkers associated with obesity from 40 fatty acids in the blood; correlation analysis was conducted between the screened potential biomarkers FAs and the chronic inflammation index hs-CRP to identify FA biomarkers associated with chronic inflammation. Changes in the fatty acid receptor CD36, inflammatory receptor TLR4, and inflammatory nuclear transcription factor NF-κB in PBMC subsets were used to further test the relationship between fatty acids and the inflammatory state in individuals with obesity. **Results**: 23 potential FA biomarkers for obesity were screened, eleven of the potential obesity biomarkers were also significantly related to hs-CRP. Compared to the control group, in monocytes the obesity group expressed higher TLR4, CD36, and NF-κB in lymphocytes, the obesity group expressed higher TLR4 and CD36; and in granulocytes the obesity group expressed higher CD36. **Conclusion**: Blood FAs are associated with obesity and are associated with chronic inflammation through increased CD36, TLR4, and NF-κB in monocytes.

Key words: Obesity; Chronic inflammation; Fatty acids

1 Introduction

In the past 40 years, obesity and related diseases have increased dramatically, and 39 % and 13 % of adults worldwide are now overweight and obese [1]. Obesity is related to the risk of type 2 diabetes, nonalcoholic fatty liver, cardiovascular disease, tumors, and other diseases [2, 3]. Chronic inflammation plays a key role in the occurrence and development of obesity-related diseases [4, 5]. Understanding the mechanisms involved in obesity-related chronic inflammation is critical for prevention of obesity-related diseases.

Chronic inflammation is closely related to the ability of immune cells to adapt to the body's needs [6, 7]. When nutrition is sufficient or energy is in surplus, the body accumulates energy, and immune cells react to this by altering their activity [8]. Thus, energy accumulation is closely related to obesity-related chronic inflammation. From the perspective of energy accumulation, excess energy is readily converted into fatty acids (FAs), which are synthesized into triglycerides and stored in adipose tissue. Despite the fact that fatty tissue has the capability to store triglycerides, its capacity to do so is constrained. The adipose tissue of obese individuals contains excessive triglycerides, resulting in a limited ability to absorb further FAs, resulting in an "overflow" into the blood, and higher concentrations of circulating FAs [9]. These FA changes not only offer potential biomarkers but also modulate the body's immune function.

High concentrations of FAs in the blood cause ectopic fat accumulation in non-fat organs that harm health and trigger chronic inflammation by stimulating immune cells [10]. There are many kinds of FAs in the blood, including saturated FAs, monounsaturated FAs, polyunsaturated FAs, and others. FAs have different functions

that are related to their chemical structure. For example, long-chain saturated FAs can cause an inflammatory activation in cultured mononuclear macrophages[11], while polyunsaturated FAs have anti-inflammatory effects [12]. Changes in the content of various blood FAs affect the physiological function of the body[13]. However, how the profile of various fatty acids in the blood of obese individuals is associated with chronic inflammation remains unclear[14]. Understanding these relationships would help guide the development of targeted interventions for obesity-related chronic inflammation and the subsequent prevention of related diseases.

This study hypothesized that high concentrations of FAs in the blood are related to chronic inflammation, and immunophenotype peripheral of PBMC involved in obesity related chronic inflammation. The biomarkers associated with obesity were identified from 40 fatty acids in the blood, and analyze the relationship between the biomarkers and chronic inflammation. Furthermore, by analyzing the difference in the expression of CD36, TLR4 and NF-κB in PBMC between obese and standard weight people, understand that immunophenotype PBMC is associated with chronic inflammation.

2 Materials and methods

2.1 Participants

Participants were recruited from the Yangzhou Lipan weight loss training camp from May 2020 to July 2020. Participants were divided into a normal weight group or obesity group. All participants were aged 18-45 years. Individuals in the normal weight group were required to have a BMI of 18.5–23.9, while the obesity group had a BMI of ⩾ 28 (The Chinese BMI criteria[15, 16]). We excluded individuals with inflammatory events, infectious diseases, long-term medication, hypertension, diabetes, professional physical training, and sports contraindications. During training, the health records of

the participants were obtained. This study was approved by Fujian Normal University with clinical trial registration number ChiCTR2200058959. The participants signed an informed consent form.

2.2 Outcome measures

We collected basic demographic information (name, gender, age, height, weight, and health history [physical examination, disease, medication, and sports injury]), BMI, blood lipid and chronic inflammation indicators, blood fatty acid content, and the expression of PBMC subsets surface receptor CD36, inflammatory receptor TLR4, and inflammatory transcription factor NF-κB.

The blood lipids of interested included total cholesterol (TC), total glyceride (TG), high-density lipoprotein cholesterol (HDLC), and Low-density lipoprotein cholesterol (LDLC). We also measured high-sensitivity C-reactive protein (hs-CRP) as a marker of chronic inflammation. Blood was collected from volunteers on an empty stomach at 7:00-8:00 in the morning the day after they joined the camp (fasting from 22:00). 5ml of blood was collected from a vein in the antecubital fossa using a vacuum tube containing a serum gel separator. The blood coagulated at room temperature for 30 min, was centrifuged at 3000 rpm for 5 min, and extract supernatant (serum) was stored in Sterile centrifuge tube, stored at 4°C. The serum samples were submitted to Nanjing Aidikang Medical Laboratory Center on the same day for analyses of TC, TG, HDLC, LDLC, and hs-CRP.

The concentrations of C4:0, C6:0, C8:0, C10:0, C11:0, C12:0, C13:0, C14:0, C14:1N5, C15:1N5, C15:0, C16:1N7, C16:0, C17:1N7, C17:0, C18:1TN9, C18:1N9, C18:2TTN6, C18:3N6, C18:3N3, C18:0, C20:1N9, C20:2N6. C20:0, C20:3N6, C20:4N6, C20:3N3, C21:0, C22:0, C23:0, C24:0, C20:5N3, C22:2N6, C22:1N9, C22:4N6, C22:5N6, C22:5N3, C22:6N3,C24:1N9 (The fatty acids corresponding to the

abbreviations are shown in Supplementary Table 1) in the serum samples were tested by an Agilent 7890/5975C gas mass spectrometer (Agilent, USA). Total saturated fatty acids (SFA) was the sum of SFAs. Total monounsaturated fatty acid (MUFA) was the sum of MUFAs. Total polyunsaturated fatty acid (PUFA) was the sum of PUFAs. Total-N3 was the sum of N3PUFAs. Total-N6 was the sum of N6PUFAs. FA concentrations were determined by Applied Protein Technology (APTBIO).

CD36, TLR4, and NF-κB p65 in PBMC subsets were tested by flow cytometry. The volunteers provided fasting blood as outlined previously into a heparin sodium tube, this was preserved at 4°C, and PBMCs were extracted within 2 hours.

CD36, TLR4, and NF-κB p65 staining and flow cytometry was as follows. NF-κB Fluorescent labeled antibody was purchased from CST, NF-κB p65 (D14E12) XP Rabbit mAb (Alexa Fluor 488 Conjugate), CD36 fluorescent labeled antibody was purchased from Biogene (APC anti human CD36 Antibody), TLR4 was purchased from BD bioscience (Hu TLR4 (CD284) PE TF901), and erythrocyte lysate, permeabilizing buffer, and fixative were purchased from Shanghai Yisheng Biotechnology Co., Ltd.

CD36 and TLR4 were stained in 100 μL of cell suspension with 5μL of fluorescent-labeled antibody in the dark at 4°C for 15min, centrifuged x5min at 500g, washed with PBS once, and resuspended in PBS for analysis. NF-κB was stained in the fixed and permeabilized cell suspension (350μL of 4°C fixative buffer, washed and centrifuged as above, 1ml of permeabilizing buffer at room temperature for 10min, washed) in 70μl of permeabilizing buffer with 1.5μl of NF-κB antibody in the dark for 30min, washed and resuspended as above. Data were acquired on a BD FACS Aria III (APC on FL4, PE on FL2, and NF- κB-FITC on FL1) and analyzed with Flowjo 10.6.2 software. Gating was for single cells (forward scatter, FSC and side scatter, SSC), then using FSC-a vs FSC-h, the mononuclear cells were selected while doublets were

excluded. Based on cell size and granularity, the granulocytes, lymphocytes, and monocytes were gated[17]. Subgrouped cell populations were then tested for TLR4-PE, CD36-APC, and NF-κB-FITC signals, with gating for positive expression being set above unstained control cells, and percentages of negative and positive populations were then recorded. The percentage of CD36 or TLR4, or NF-κB cells was determined by calculating the difference between the percentage of positive cells in the test vial and that in the control vial.

2.3 Statistical analysis

The screening of potential biomarkers of obesity was completed by SIMCA-P 14.1 software. The fatty acid content data matrix was imported into SIMCA-P 14.1 software for unsupervised principal components analysis (PCA) and supervised orthogonal partial least squares discriminant analysis (OPLS-DA)[18]; the principal component with the largest contribution to inter-group variability was identified through the OPLS-DA model. The permutation experiment and CV-ANOVA method further verified the validity of the model. If the R^2Y and Q^2 values of the model after random permutation were both less than the model values, the actual model was deemed to be valid. The CV-ANOVA gain at $P<0.05$ indicated that the model was established successfully. The variable importance in projection (VIP) of each fatty acid variable was obtained according to the OPLS-DA model analysis, and the potential biomarkers were screened by using VIP>1.0 as the standard [19]. The VIP of fatty acid >1.0, and the mean comparison of fatty acid content between the obesity group and the normal weight group ($P<0.05$) were used as the criteria for screening potential biomarkers of obesity.

Data are expressed as mean ± standard deviation and compared by independent samples t-tests. Statistical Package R (The R Foundation, version 3.1.2) and Empower (R) were used for these analyses. $P < 0.05$ and $p < 0.01$ indicate statistically significant

differences.

2.4 Evaluation of sample size

The discriminant analysis model was used to estimate the required study sample size. We determined that at n = 50, the specificity and sensitivity of discrimination could reach 0.85[20]. The actual sample size was 52 individuals, including 25 (male:60%) in the normal weight group and 27 (male: 59.26%) in the obesity group, which meets the sample size requirements.

3 Results

3.1 Study population characteristics

25 control individuals (normal weight group) and 27 obese individuals (Obesity group) were enrolled. Study participant clinical characteristics are shown in Table 1. There were no significant differences in age or height between the normal weight and the obesity group. There were significant differences in weight, BMI, TC, TG, HDLC, LDLC, and hs-CRP between the two groups.

Table 1. Participant characteristics by study group. Significant differences are indicated in bold.

	Normal weight N=25	Obesity N=27	*P*
female	10 (40.00%)	11 (40.74%)	0.956
male	15 (60.00%)	16 (59.26%)	
Age (years)	27.40 ± 6.55	30.19 ± 5.71	0.108
Height (cm)	167.08 ± 6.93	170.19 ± 8.32	0.152
Weight (kg)	62.10 ± 5.58	102.69 ± 21.97	**<0.001**
BMI (kg/m2)	22.22 ± 1.53	35.11 ± 5.05	**<0.001**
TC(mmol/L)	4.32 ± 0.58	4.96 ± 1.00	**0.007**
TG(mmol/L)	0.99 ± 0.47	1.78 ± 1.17	**0.003**

HDLC(mmol/L)	1.33 ± 0.16	1.19 ± 0.22	**0.011**
LDLC(mmol/L)	2.50 ± 0.51	3.08 ± 0.70	**0.001**
hs-CRP(mg/L)	1.64 ± 0.69	4.87 ± 1.96	**<0.001**

3.2 Fatty acid concentrations in blood

The concentration of fatty acids is shown in Table 2. This study found no compared differences in serum C10:0, C12:0, C18:3N6, C20:0, C22:1N9, C22:2N6, C23:0, or C24:0 between the two groups, but the concentration of other 31 FAs, total SFA, total MUFA, total PUFA, total N3, and total N6 were higher in the obesity group compared to the normal weigh group.

Table 2. Comparison of blood FAs by group. Significant differences, VIP > 1 are indicated in bold and reflect potential obesity biomarkers.

fatty acid	Normal weigh （N=25）	Obesity （N=27）	*P*	*VIP*	potential biomarkers
Total-SFA	423.22 ± 75.96	619.78 ± 228.77	**<0.001**	**1.294**	Yes
C6:0	0.00 ± 0.00	0.01 ± 0.00	**0.007**	0.961	
C8:0	0.26 ± 0.11	0.49 ± 0.38	**0.005**	0.985	
C10:0	0.74 ± 0.39	0.81 ± 0.46	0.562	0.562	
C11:0	0.00 ± 0.00	0.01 ± 0.00	**0.003**	0.945	
C12:0	0.44 ± 0.35	0.60 ± 0.57	0.244	0.669	
C13:0	0.01 ± 0.00	0.03 ± 0.02	**0.002**	**1.101**	Yes
C14:0	4.29 ± 3.09	11.29 ± 12.51	**0.009**	**1.100**	Yes
C15:0	1.23 ± 0.48	2.00 ± 0.98	**<0.001**	**1.180**	Yes
C16:0	285.51 ± 53.32	428.24 ± 157.92	**<0.001**	**1.304**	Yes
C17:0	2.26 ± 0.80	3.49 ± 1.60	**0.001**	**1.106**	Yes
C18:0	109.16 ± 24.02	151.24 ± 54.64	**<0.001**	**1.140**	Yes
C20:0	0.74 ± 0.32	0.95 ± 0.63	0.143	0.637	

C21:0	0.12 ± 0.03	0.17 ± 0.07	**0.008**	0.995	
C22:0	9.25 ± 3.50	15.61 ± 8.03	**<0.001**	**1.137**	Yes
C23:0	0.05 ± 0.01	0.07 ± 0.03	0.074	0.700	
C24:0	0.27 ± 0.09	0.34 ± 0.20	0.088	0.619	
Total-MUFA	283.69 ± 85.00	487.26 ± 227.64	**<0.001**	**1.268**	Yes
C14:1N5	0.13 ± 0.15	0.69 ± 0.79	**<0.001**	0.965	
C15:1N5	0.39 ± 0.28	0.77 ± 0.43	**<0.001**	**1.145**	Yes
C16:1N7	13.37 ± 6.15	36.47 ± 27.30	**<0.001**	**1.247**	Yes
C17:1N7	0.85 ± 0.35	2.00 ± 1.24	**<0.001**	**1.298**	Yes
C18:1TN9	0.32 ± 0.13	0.45 ± 0.21	**0.014**	0.753	
C18:1N9	216.34 ± 57.46	393.59 ± 185.98	**<0.001**	**1.306**	Yes
C20:1N9	2.17 ± 0.56	3.35 ± 1.64	**0.001**	**1.020**	Yes
C22:1N9	0.98 ± 0.64	1.21 ± 0.94	0.305	0.591	
C24:1N9	30.44 ± 10.19	45.64 ± 18.03	**<0.001**	**1.048**	Yes
Total-PUFA	643.26 ± 151.60	795.67 ± 254.27	**0.012**	**1.016**	Yes
Total-N3	28.88 ± 7.77	40.92 ± 19.09	**0.005**	**1.126**	Yes
C18:3N3	9.43 ± 4.61	14.47 ± 6.90	**0.003**	0.982	
C20:3N3	0.91 ± 0.27	1.07 ± 0.40	0.104	0.809	
C20:5N3	0.24 ± 0.07	0.40 ± 0.21	**<0.001**	**1.167**	Yes
C22:5N3	8.45 ± 2.22	12.34 ± 4.82	**<0.001**	**1.122**	Yes
C22:6N3	8.40 ± 2.65	11.12 ± 4.04	**0.006**	**1.046**	Yes
Total-N6	614.37 ± 146.87	754.75 ± 237.08	**0.014**	0.997	
C18:2TTN6	0.06 ± 0.03	0.11 ± 0.09	**0.022**	0.676	
C18:2N6	392.81 ± 86.37	517.29 ± 178.12	**0.003**	**1.098**	Yes
C18:3N6	10.08 ± 11.95	5.42 ± 2.76	0.055	0.554	
C20:2N6	5.04 ± 1.41	6.60 ± 2.66	**0.012**	**1.034**	Yes
C20:3N6	22.95 ± 11.17	32.75 ± 15.90	**0.014**	0.921	

C20:4N6	137.72 ± 42.51	179.85 ± 57.52	**0.004**	0.894	
C22:2N6	0.18 ± 0.07	0.23 ± 0.20	0.179	0.597	
C22:4N6	4.90 ± 1.13	6.57 ± 2.40	**0.003**	**1.028**	Yes
C22:5N6	4.83 ± 1.23	5.89 ± 2.02	**0.029**	0.775	

3.3 Obesity accompanied by high concentrations of free fatty acids in blood

Potential biomarkers of obesity were screened. The FAs in the two groups were analyzed by PCA. It can be seen from the PCA score chart that FAs in the obese and normal weight groups were well differentiated in the chart (Supplementary Fig. 1-a), suggesting that there were variations in the profiles of fatty acids between the two groups.

By analyzing the score plot of the OPLS-DA model (Supplementary Fig. 1-b), it can be seen that the FA scores of the obese and normal weight groups are clustered, with perfect separation, no overlap, and almost no crossover, Q2=0.49>0.4, R2Y=0.713, which shows that the model has good goodness of fit and predictive power.

The CV-ANOVA analysis results showed that the OPLS-DA model was significant (P<0.01). The random array experiment carried out 200 array experiments on the model[21]. After the random array, the R2Y and Q2 values of the OPLS-DA model were smaller than the actual model values (Supplementary Fig. 1-c), which shows that the model was effective.

The VIP value of each FA obtained by the OPLS-DA model and the difference test results of the average fatty acid content between the two groups are shown in Table 2. Among many FAs, the following conditions were required when screening potential biomarkers of obesity: in the OPLS-DA model, the contribution index VIP of fatty acid evaluation and discrimination effectiveness is >1.0 and there is a significant difference

in the mean value of fatty acid content between the two groups. In this study, C17:1N7, C15:0, C17:0, C14:0, C18:2N6, C20:2N6, C18:1N9, C16:0, C16:1N7, C20:5N3, C15:1N5, C18:0, C22:0, C22:5N3, C13:0, C24:1N9, C22:6N3, C22:4N6, C20:1N9, total PUFA, total SFA, total MUFA, and total N3 all met these requirements.

3.4 The relationship between potential biomarkers and chronic inflammation

This study used hs-CRP as an indicator of chronic inflammation and analyzed its relationship with FAs, found that C20:4N6, C15:1N5, C18:1N9, C11:0, C20:5N3, C16:0, total SFA, total-N6, C13:0, C18:0, C24:1N9, C21:0, C22:0, C22:4N6, C17:1N7, and C24:0 significantly related to hs-CRP (Table 3).

Table 3. Correlation between fatty acids and hs-CRP

fatty acid	*R*	*P*	fatty acid	*R*	*P*	fatty acid	*R*	*P*
C20:4N6	0.3768	**0.0059**	C20:1N9	0.2678	0.0549	C14:0	0.1828	0.1946
C15:1N5	0.3582	**0.0091**	C16:1N7	0.2666	0.0561	C20:3N6	0.1648	0.2429
C18:1N9	0.3510	**0.0107**	C6:0	0.2572	0.0657	C18:1TN9	0.1533	0.2779
C11:0	0.3303	**0.0168**	C23:0	0.2536	0.0697	C22:1N9	0.1521	0.2818
C20:5N3	0.3298	**0.0170**	C8:0	0.2415	0.0845	C18:3N6	-0.1457	0.3027
C16:0	0.3264	**0.0182**	C22:5N3	0.2414	0.0847	C20:2N6	0.1346	0.3413
Total-SFA	0.3152	**0.0229**	C22:6N3	0.2357	0.0925	C22:2N6	0.1337	0.3448
Total-N6	0.3128	**0.024**	C18:2N6	0.2330	0.0964	C20:0	0.1223	0.3878
C13:0	0.3123	**0.0242**	C14:1N5	0.2306	0.1000	C20:3N3	0.1120	0.4292
C18:0	0.3051	**0.0278**	C17:0	0.2269	0.1057	C18:2TTN6	0.0573	0.6868
C24:1N9	0.3036	**0.0287**	C15:0	0.2242	0.1100	C10:0	-0.0419	0.768
C21:0	0.2883	**0.0382**	C22:5N6	0.2200	0.1172	C12:0	0.0232	0.8703
C22:0	0.2881	**0.0383**	Total-PUFA	0.2137	0.1282			
C22:4N6	0.2878	**0.0385**	C18:3N3	0.2134	0.1288			
C17:1N7	0.2839	**0.0414**	Total-N3	0.2116	0.1321			

C24:0	0.2753	**0.0482**	Total-MUFA	0.2110	0.1333	

correlation coefficient: *R*

Based on these data, this study found that C17:1N7, C18:1N9, C16:0, C20:5N3, C15:1N5, C18:0, C22:0, C13:0, C24:1N9, C22:4N6, and total SFA are possible bridges that connect obesity and chronic inflammation (Fig. 1).

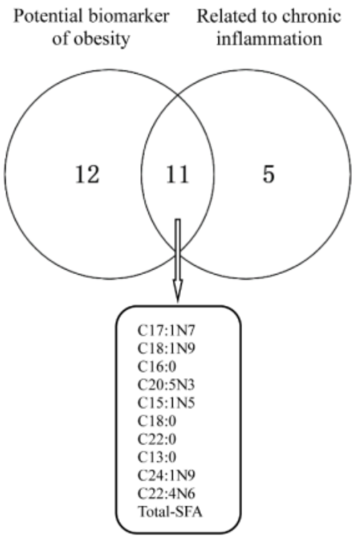

Figure 1 Intersection between potential biomarkers of obesity and fatty acids related to chronic inflammation.

3.5 Expression of CD36, TLR4, and NF-κB in PBMC subsets

To further understand the relationship between FAs and chronic inflammation, nine individuals from the normal weight group and the obesity group were respectively randomly selected for exploratory research. Peripheral blood was collected to obtain PBMCs, and the expression of CD36, TLR4 (Fig 2), and NF-κB (Supplementary Fig 2)

in PBMC subsets (monocytes, lymphocytes, and granulocytes) was analyzed by flow cytometry.

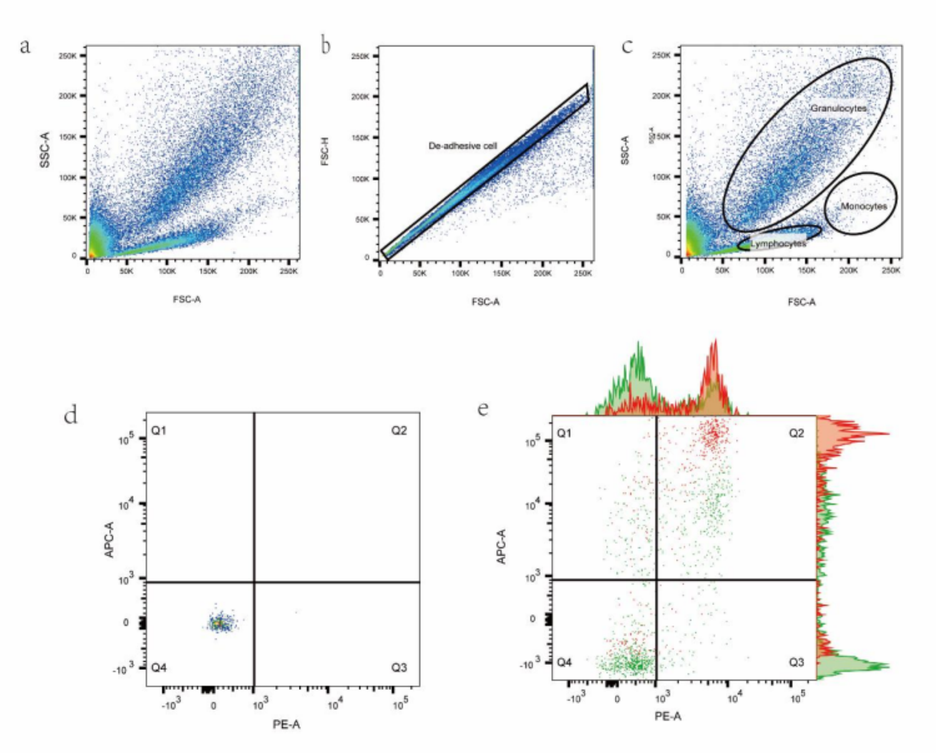

Figure 2. Gating strategy. (a and b) Gating for single cells, (c) identification of PBMC subsets based on size and granularity, (d) quantification of CD36, TLR4 signals in each cell subtype with positivity determined based on an unstained sample, and (e) proportion of TLR4 and CD36 positive monocytes in obesity and standard weight, red: obesity group, green: normal weight group.

This study found that (Table 4), compared to the normal weight group, the obesity group expressed higher TLR4, CD36, and NF-κB in monocytes; the obesity group expressed higher TLR4 and CD36 in lymphocytes; and the obesity group expressed higher CD36 in granulocytes.

Table 4. Proportion cells of CD36, TLR4, and NF-κB in PBMC subset.

The percentage of CD36 or TLR4, or NF-κB cells in PBMC subset (%).

PBMC subsets		Normal weigh (N=9, male:55.56%)	Obesity (N=9, male:55.56%)	*P*
Monocytes	CD36	42.8 ± 06.3	75.9 ± 11.1	**<0.001**
	TLR4	50.5 ± 07.6	67.4 ± 12.3	**0.003**
	NF-κB	9.1 ± 7.0	21.2 ± 12.8	**0.027**
Lymphocytes	CD36	11.5 ± 4.3	18.0 ± 6.6	**0.024**
	TLR4	3.3 ± 1.3	6.5 ± 2.6	**0.004**
	NF-κB	2.2 ± 2.5	2.7 ± 1.8	0.627
Granulocytes	CD36	23.9 ± 12.0	55.0 ± 23.5	**0.003**
	TLR4	5.6 ± 3.3	3.9 ± 1.8	0.206
	NF-κB	21.5 ± 19.9	29.5 ± 23.2	0.456

4 Discussion

4.1 Chronic inflammation severity is related to higher blood FAs in obese individuals

FAs provide a sensitive index of disordered lipid metabolism [22]. Blood FAs include non-esterified FAs and esterified FAs bound to triglycerides. Blood FAs are composed of exogenous FAs from dietary sources and endogenous FAs synthesized in the body. Many diseases, such as cardiovascular diseases, tumors, and metabolic diseases, can show abnormal FAs profiles in the blood [23-25]. Our study shows that 31 FAs, total SFA, total PUFA, total MUFA, total N3, and total N6 are higher in obese individuals (Table 2). With the high fat and energy intake, once FAs are utilized to maintain physiological function, the surplus remains in the circulation resulting in higher measurable FA concentrations. As such, blood FAs are potential biomarkers of obesity.

We utilized Agilent 7890/5975C gas mass spectrometer (Agilent, USA) to detect 39 out of our target 40 FAs (C4:0 was not detected) in serum. Potential biomarkers associated with obesity were identified including C17:1N7, C15:0, C17:0, C14:0, C18:2N6, C20:2N6, C18:1N9, C16:0, C16:1N7, C20:5N3, C15:1N5, C18:0, C22:0, C22:5N3, C13:0, C24:1N9, C22:6N3 C22:4N6, C20:1N9, total PUFA, total SFA, total MUFA, and total-N3, through PCA and OPLS-DA statistical strategies. These markers include SFAs, MUFAs, and PUFAs.

When basic physiological requirements are fulfilled, excess energy is stored in the form of fat that ultimately increases body size and weight. We show that both protective and harmful FA concentrations are increased in obese individuals. This suggests that during energy surplus, FAs are stored without preference for their function and their abundance is reflected in a higher blood FA concentration. In addition to reflecting the energy surplus, high blood FA concentrations also play other roles, specifically in chronic inflammation.

This study screened FAs as a potential biomarker of obesity and correlated them with hs-CRP, a chronic inflammatory marker, found that C17:1N7, C18:1N9, C16:0, C20:5N3, C15:1N5, C18:0, C22:0, C13:0, C24:1N9, C22:4N6, and total SFA were both potential biomarkers of obesity and significantly associated with chronic inflammation. These FAs may partly explain the relationship between obesity and chronic inflammation. Obese individuals have rich fat stores. When excess fat cannot enter lipid droplets, it results in a chronic increase in blood FAs. The connection between FAs and chronic inflammation can be analyzed from the fatty acid receptors on the surface of cells and inflammatory transcription factors in the nucleus [26]. Research has shown that excess FAs can further activate pro-inflammatory transcription factors in the nucleus by interacting with various receptors such as CD36 [27] and TLR4 [28],

thereby affecting the inflammatory state of cells. However, the influence of FAs on cellular inflammatory pathways may differ among different cells [7]. This study starts with the inflammatory phenotype of PBMC cell subpopulations and analyzes the impact of fatty acids on cellular inflammatory signaling pathways.

4.2 Increased FAs were associated with increased CD36, TLR4, and NF-κB in monocytes in obese individuals.

The proinflammatory effects of blood FAs can be determined by assessing for immune cell phenotype differences. The entry of FAs, especially long-chain FAs, into cells requires the coordination of cell surface receptors. CD36 is a scavenger receptor with a high affinity for long-chain FAs, which can help cell internalization of FAs. Increases in its expression are closely related to high concentrations of FAs [29-31]. Moreover, CD36 has a variety of ligands and signal transduction capabilities and can participate in inflammation [32, 33].

TLR4 is an immune-related cell surface receptor that can link excess nutrition with inflammation. CD36 participates in TLR4-dependent inflammatory responses induced by various ligands [34-36]. CD36 plays an important role in helping TLR4 recognize LPS, ox LDLC, long-chain fatty acids, and other ligands to transmit signals to cells [37]. When TLR4 recognizes ligand transmission signals on the cell surface, CD36 can form a polymer with TLR4 through tyrosine kinase to amplify the downstream proinflammatory signal pathway [38, 39]. MyD88, TRIF downstream of TLR4 is activated to promote the expression and secretion of the NF-κB inflammatory pathway [40, 41]. Changes in CD36, TLR4, and NF-κB expression are related to high concentrations of extracellular FAs.

This study showed that the blood concentrations of FAs were higher in the obesity group and that this was related to chronic inflammatory. However, the relationship

between the pro-inflammatory effect of fatty acid receptors on immune cells and obesity requires further investigation. In this study, we randomly selected 9 participants to explore the differences in PBMC inflammatory phenotypes between obese and non-obesity group, find that CD36 and TLR4 were increased on the surface of lymphocytes and granulocytes in the obesity group, but this was not associated with changes in intracellular NF-κB. On the other hand, in monocytes, increases in CD36 and TLR4 were associated with increased NF-κB in the cells. Changes in CD36 and TLR4 on monocytes may lead to the secondary signal transductions that activate NF-κB, increasing the expression of downstream inflammatory genes, a pro-inflammatory cell phenotype, the promotion of chronic inflammation.

The mechanisms of obesity-associated chronic inflammation are complex, and currently tissue hypoxia, oxidative stress, endoplasmic reticulum stress, insulin resistance, and gut microbial changes are among the pathways that can explain obesity-associated chronic inflammation [42]. This study synthesized multiple mechanisms mentioned above and found that most of them are related to energy metabolism and immune cell function. Therefore, the relationship between changes in energy metabolism and the pro-inflammatory state of immune cells in obese individuals may be an important idea to understand the mechanisms of obesity-associated chronic inflammation [43]. This study found that high concentrations of fatty acids, potential markers of obesity, in the blood of obese individuals may be responsible for chronic inflammation, while different immune phenotypes of PBMC cell subpopulations may be a potential mechanism chronic inflammation induced by obesity. The present study analyzed the potential mechanism from the perspective of the relationship between energy accumulation and immune cells, however, its molecular mechanism needs to be investigated in depth.

Comparisons with other studies and what does the current work add to the existing knowledge

Among the 23 potential biomarkers of obesity, 11 biomarkers were also significantly associated with chronic inflammation, including C17:1N7, C18:1N9, C16:0, C20:5N3, C15:1N5, C18:0, C22:0, C13:0, C24:1N9, C22:4N6, and total SFA.

Study strengths and limitations

This pilot study's strength lies in its novel and interesting findings, but its main limitation is the small sample size. Since the study is observational, it's only possible to establish a correlation between fatty acids, immune phenotypes of PBMC and chronic inflammation based on correlation analyses or difference comparisons. Therefore, it's essential to conduct larger prospective trials in the future to establish potential causality.

5 Conclusions

23 potential biomarkers of obesity were identified by screening for serum fatty acids. These biomarkers were more highly concentrated in obese individuals. Within a certain range, the concentrations of these potential biomarkers were associated with increases in obesity. Among the 23 potential biomarkers of obesity, 11 biomarkers were also significantly associated with chronic inflammation. The high expression of CD36, TLR4, and NF-κB in monocytes may be involved in chronic inflammation caused by obesity. In the future, Additional studies must be conducted in larger populations, with primary endpoints based on clinical events, to determine whether estimated blood lipid profiles and immune phenotypes of PBMC subsets can be used widely in the clinical care of patients with obesity.

Acknowledgments

The authors thank EditSprings (https://www.editsprings.cn) for revising the manuscript and polishing the language.

Declarations

Ethical Approval

The trial received ethical approval from Fujian Normal University and was assigned clinical trial registration number ChiCTR2200058959. The participants signed an informed consent form, and all the methods employed were conducted in compliance with the applicable Declaration of Helsinki.

Competing interests

We have no competing interests.

Authors' contributions

SL and CH designed the study; LF and QM was responsible for Collecting sample and testing indicators; SL, YY and LF performed the statistical; SL and CH contributed to interpreting the results; SL wrote the first manuscript draft; SL, CH, LF, QM, YY contributed to the final manuscript. All authors have read and agreed to the published version of the manuscript.

Funding

The funded was provided by National Natural Science Foundation of China (31971099) and Jiangsu Federation of philosophy and Social Sciences(21WRA001).

Availability of data and materials

The datasets are available from the corresponding author.

Supplementary Figure 1. Screening of potential biomarkers of obesity, (a) PCA (green: Normal weigh, blue: Obesity), (b) OPLS-DA (green: Normal weigh, blue: Obesity), (c) model of random array experiment (n: 200, green: R^2, blue: Q^2).

Supplementary Figure 2. Gating strategy. (a) use the control cells without antibody as the control, define the range of negative cells (NF- κB marked with FITC), (b) Proportion of NF-κB p65 positive monocytes in normal weight, and (c) Proportion of

NF-κB positive monocytes in obesity. PBMC subsets (Monocytes, Lymphocytes, Granulocytes) were determined by combining cell size and the number of cellular particles to determine their position, the gating strategy of NF-κB p65 is the same as that of CD36 and TLR4.

Supplementary Table 1. The fatty acids corresponding to the abbreviations.

Peripheral blood mononuclear cells (PBMC) involved in obesity related chronic inflammation caused by free fatty acids

ORIGINALITY REPORT

12%
SIMILARITY INDEX

PRIMARY SOURCES

1	www.ncbi.nlm.nih.gov<br>Internet	81 words — 1%
2	www.mdpi.com<br>Internet	40 words — 1%
3	Lisa, M.. "High-performance liquid chromatography-atmospheric pressure chemical ionization mass spectrometry and gas chromatography-flame ionization detection characterization of @D5-polyenoic fatty acids in triacylglycerols from conifer seed oils", Journal of Chromatography A, 20070330<br>Crossref	39 words — 1%
4	Algae for Biofuels and Energy, 2013.<br>Crossref	34 words — 1%
5	www.science.gov<br>Internet	33 words — 1%
6	Ya-Jie Xu, Wen-Juan Wang, Qiu-Yi Zhang, Meng-Nan Yang et al. "Docosahexaenoic acid supplementation in gestational diabetes mellitus and neonatal metabolic health biomarkers", Frontiers in Nutrition, 2023<br>Crossref	26 words — < 1%

7	Christopher Papandreou, Aleix Sala-Vila, Serena Galié, Jananee Muralidharan et al. "Association Between Fatty Acids of Blood Cell Membranes and Incidence of Coronary Heart Disease", Arteriosclerosis, Thrombosis, and Vascular Biology, 2019<br>Crossref	25 words — < 1%
8	McGinley, Edwa. "Estimation of striped bass (Morone saxatilis) diets using fatty acid signature analysis", Proquest, 2014.<br>ProQuest	19 words — < 1%
9	www.pubfacts.com<br>Internet	19 words — < 1%
10	hdl.handle.net<br>Internet	18 words — < 1%
11	proceedings.science<br>Internet	17 words — < 1%
12	Jia-Xing Zhang, Wen Li, Xiu-Juan Tao, Chen Chen et al. "Fat-to-muscle ratio as a predictor for dyslipidaemia in transitional-age youth", Lipids in Health and Disease, 2022<br>Crossref	16 words — < 1%
13	www.dovepress.com<br>Internet	16 words — < 1%
14	www.researchgate.net<br>Internet	16 words — < 1%
15	Meng-Jiao Sun, Bing-Hu Li, Chun-Yan Long, Yan-Qin Wang et al. "Association between serum uric acid levels and cerebral white matter lesions in Chinese individuals", International Journal of Neuroscience, 2016	14 words — < 1%

Crossref

16 www.researchsquare.com
Internet
14 words — < 1%

17 Marjan Mahdavi-Roshan, Arsalan Salari, Azin Vakilpour, Amir Savar Rakhsh, Zeinab Ghorbani. "Rice bran oil could favorably ameliorate atherogenicity and insulin resistance indices among men with coronary artery disease: post hoc analysis of a randomized controlled trial", Lipids in Health and Disease, 2021
Crossref
13 words — < 1%

18 ir.nctu.edu.tw
Internet
13 words — < 1%

19 iai.asm.org
Internet
12 words — < 1%

20 journals.lww.com
Internet
12 words — < 1%

21 pdfs.semanticscholar.org
Internet
12 words — < 1%

22 www.cellsignal.com
Internet
12 words — < 1%

23 epub.ub.uni-muenchen.de
Internet
11 words — < 1%

24 Qianqian Zhang, Xuemei Li, Xiaoxin Gao, Chunran Cao, Yuchi Hu, Hongzhu Guo. " Total saponins from stems and leaves of L. ameliorate podophyllotoxin ‐ induced myelosuppression and gastrointestinal toxicity ", Biomedical Chromatography, 2021
Crossref
10 words — < 1%

25 thieme-connect.com
Internet
10 words — < 1%

26 storage.googleapis.com
Internet
9 words — < 1%

27 www.angabinteb.com
Internet
9 words — < 1%

28 www.ijlpr.com
Internet
9 words — < 1%

29 Hoffman, L.C.. "The effects of region and gender on the fatty acid, amino acid, mineral, myoglobin and collagen contents of impala (Aepyceros melampus) meat", Meat Science, 200503
Crossref
8 words — < 1%

30 Matthias Schreiner. "Optimization of Solvent Extraction and Direct Transmethylation Methods for the Analysis of Egg Yolk Lipids", International Journal of Food Properties, 2006
Crossref
8 words — < 1%

31 Tao Xie, Yinuo Liu, Huixian Lu, Ambreen Iqbal, Mengru Ruan, Ping Jiang, Haibin Yu, Jilun Meng, Zhihui Zhao. "The Knockout of the ASIP Gene Altered the Lipid Composition in Bovine Mammary Epithelial Cells via the Expression of Genes in the Lipid Metabolism Pathway", Animals, 2022
Crossref
8 words — < 1%

32 Xiaojiao Yi, Junfeng Zhu, Jinghui Zhang, Yun Gao, Zhongjian Chen, Shihai Lu, Zongwei Cai, Yanjun Hong, Yongjiang Wu. "Investigation of the reverse effect of Danhong injection on doxorubicin-induced cardiotoxicity in H9c2 cells: Insight by LC–MS based non-targeted metabolomic
8 words — < 1%

analysis", Journal of Pharmaceutical and Biomedical Analysis, 2018
Crossref

33 e-century.us
Internet
8 words — < 1%

34 ro-journal.biomedcentral.com
Internet
8 words — < 1%

35 www.frontiersin.org
Internet
8 words — < 1%

36 Andrea de la Garza Puentes, Rosa Montes Goyanes, Aida Maribel Chisaguano Tonato, Ana Isabel Castellote et al. "Evaluation of less invasive methods to assess fatty acids from phospholipid fraction: cheek cell and capillary blood sampling", International Journal of Food Sciences and Nutrition, 2015
Crossref
7 words — < 1%

37 Lin-Lin Pan, Hsiu-Chen Yu, Ching-Hui Lee, Kuo-Chuan Hung, I-Ting Tsai, Cheuk-Kwan Sun. "Impact of Staining Methods and Human Factors on Accuracy of Manual Reticulocyte Enumeration", Diagnostics, 2022
Crossref
7 words — < 1%

38 "Social Robotics", Springer Science and Business Media LLC, 2022
Crossref
6 words — < 1%

39 Peng Shen, Qingchuan Hu, Meixue Dong, Shunjie Bai et al. "Venlafaxine exerts antidepressant effects possibly by activating MAPK–ERK1/2 and P13K–AKT pathways in the hippocampus", Behavioural Brain Research, 2017
Crossref
6 words — < 1%

40	Xiaobin Li, Chenyang Li, Yongqiang Zhu, Yongping Shi et al. "Lipid Fingerprinting of Different Material Sources by UPLC-Q-Exactive Orbitrap/MS Approach and Their Zebrafish-Based Activities Comparison", Journal of Agricultural and Food Chemistry, 2020<br>Crossref	6 words — < 1%

EXCLUDE QUOTES	OFF	EXCLUDE SOURCES	OFF
EXCLUDE BIBLIOGRAPHY	OFF	EXCLUDE MATCHES	OFF
